# Prevalence of Social Anxiety and Associated Risk Factors Among Adults in the United Arab Emirates Following the COVID-19 Lockdown

**DOI:** 10.7759/cureus.99669

**Published:** 2025-12-19

**Authors:** Raghad Al Khatib, Patol Alsabagh, Amal Abudouleh, Manar H Hussein, Abdulrahman AlAyyaf, Khalid Ali, Amal Hussein, Deepika M Kamath

**Affiliations:** 1 College of Medicine, University of Sharjah, Sharjah, ARE; 2 Family and Community Medicine, University of Sharjah, Sharjah, ARE; 3 Basic Medical Science, University of Sharjah, Sharjah, ARE

**Keywords:** anxiety disorders, covid-19 lockdown, mental health, middle east, personality traits, psychosocial impact, public health, risk factors, social anxiety, united arab emirates

## Abstract

Background

Social anxiety has been a persistent matter among adults and has peaked during the span of the COVID-19 pandemic. One of the nationwide preventive methods implemented to control the spread of the virus was quarantine, which caused isolation and issues like social anxiety. This study aims to assess social anxiety levels among adults in the United Arab Emirates (UAE) following the COVID-19 lockdown and identify major risk factors and personality traits associated with social anxiety.

Methodology

Individuals aged 18 years and above were recruited using snowball sampling methods through a cross-sectional online survey. The survey consisted of questions on demographics, COVID-19 infection, quarantine conditions, and standard scales, including the Depression, Anxiety, and Stress Scale (DASS-21) and the Ten-Item Personality Inventory (TIPI).

Results

A total of 409 participants were included in the study, with severe social anxiety observed in 81 (29.6%) of females and 22 (16.3%) of males. Severe social anxiety was significantly more prevalent among younger adults, affecting 82 individuals (33.1%) aged 18-35 compared with 21 individuals (13.0%) aged 36 years and older (*P* < 0.001). Higher prevalence was also observed among UAE nationals (*P* = 0.009), students (*P* < 0.001), and unmarried individuals (*P* < 0.001). A prior diagnosis of social anxiety was associated with an increased likelihood of severe social anxiety following the lockdown, and individuals who had contracted COVID-19 or were concerned about contracting the virus reported higher levels of social anxiety. Additionally, participants with high levels of openness to experience (18, 41.9%), emotional stability (24, 41.4%), conscientiousness (36, 39.1%), and agreeableness (21, 41.2%) demonstrated significantly greater social anxiety severity.

Conclusions

The prevalence of social anxiety is significantly high following the COVID-19 lockdown. Vulnerable groups, such as young people, women, unmarried individuals, and those with elevated levels of certain personality traits, should receive appropriate interventions.

## Introduction

The COVID-19 pandemic has significantly altered social dynamics worldwide, imposing extended periods of isolation and major lifestyle changes. Recent studies indicate that individuals subjected to isolation and quarantine often experience heightened levels of anxiety, frustration, confusion, and stress [1]. Social anxiety, defined as an excessive fear of social interactions and avoidance of social situations due to perceived negative evaluation, is exacerbated by the prolonged absence of in-person interactions during the pandemic [2].

A systematic review and meta-analysis of 38 studies estimated that Social Anxiety Disorder (SAD) affects 4.7% of children, 8.3% of adolescents, and 17% of youth, highlighting a progressive increase with age [3]. Additionally, data from the World Mental Health Survey Initiative revealed substantial cross-national differences, with lifetime prevalence rates ranging from 0.2% in Nigeria to 13.7% in the United States, emphasizing the role of cultural and environmental factors in shaping SAD prevalence [4]. These findings underscore the global burden of SAD and the need for targeted prevention and intervention strategies.

In the UAE, the prevalence of mental health is a matter of great concern. A study among adolescents aged 13-18 years reported an overall anxiety disorder prevalence of 28%, with higher rates in females (33.6%) than males (17.2%) [5]. Additionally, research conducted on Abu Dhabi residents aged 18-75 years revealed that 46.0% of participants screened positive for depression, while 37.5% exhibited moderate to severe anxiety levels [6]. A study conducted to measure anxiety levels among UAE university students additionally found that 32.2% of participants experienced moderate to severe levels of anxiety following the COVID-19 lockdown [7]. These findings suggest a preexisting vulnerability to anxiety and depressive disorders in the UAE population, which may have been exacerbated by the pandemic. To our knowledge, no prior study has specifically investigated the prevalence and associated risk factors for social anxiety in adults in the UAE post-COVID-19 lockdown. 

This cross-sectional study aims to estimate the prevalence of social anxiety among adults in the UAE post-lockdown and to identify associated risk factors such as COVID-19 lockdown impact, personality type, and certain demographics.

## Materials and methods

Study design

This cross-sectional study was conducted between February and April 2022 using the snowball sampling method. Snowball sampling was selected because the target population may be reluctant to participate in studies on mental health due to cultural stigma and sensitivity. By receiving referrals from trusted participants, this method helps reach individuals who might otherwise be inaccessible, thereby enhancing participant trust, engagement, and data reliability. The study was approved by the Ethics and Research Committee of the University of Sharjah (REC-22-02-14-03-S) on February 14, 2022.

Study sample

Cochran's formula (N = 4P(1-P)/ME^2^) was used to calculate the sample size in a cross-sectional study estimating a single proportion. N is the sample size, P is the expected prevalence, estimated at 50% (P = 0.5), and ME is the margin of error, set at 5% [8]. Using this formula, the calculated sample size was 400 participants. A total of 800 participants were recruited, of whom 409 responded fully to the survey. The study included UAE residents aged 18 and above who were Arabic or English speakers with access to social media. Individuals under 18 or non-UAE residents were excluded.

Data collection

The demographic section of the questionnaire collected essential participant information, including age, sex, ethnicity, marital status, employment status, and previous diagnosis of social anxiety. Additionally, a COVID-19-related section was included to gather information on participants’ infection status, exposure to positive cases, and experiences during lockdown. These questions aimed to explore potential changes in social anxiety levels linked to the pandemic.

The Depression Anxiety Stress Scale-21 (DASS-21), a validated psychometric tool, was utilized to assess anxiety levels within the study population [9]. Its reliability and validity have been established through rigorous methodological procedures, including factor analysis, construct validation, and internal consistency assessment using Cronbach’s alpha [10]. The scale consists of 21 items divided into three subscales: depression, anxiety, and stress, each containing seven items, each scored separately. Although the DASS-21 scale measures three distinct parameters, our research only assessed the anxiety subscale, as the other parameters were outside the study's objectives. Participants rated their experiences on a four-point Likert scale, with higher scores indicating greater severity. The final scores were classified into normal (0-7), mild to moderate (8-14), and severe to extremely severe (15+) categories based on established thresholds, providing a standardized approach to assessing anxiety levels [10].

Personality traits were evaluated using the Ten-Item Personality Inventory (TIPI), a concise measure of the Big Five personality dimensions, including agreeableness, conscientiousness, extraversion, emotional stability, and openness to experience [11]. Although the TIPI exhibits lower Cronbach’s alpha values, ranging from 0.40 to 0.73, its design prioritizes content validity over internal consistency, allowing for a rapid yet effective assessment of personality traits [12]. This questionnaire was selected due to its brevity, aligning with the study’s objective of utilizing a time-efficient and practical assessment tool.

The questionnaire was available in validated English and Arabic forms. They were distributed online via Google Forms and shared across multiple social media platforms. Participants were required to provide informed consent before completing the survey. The estimated completion time was approximately 10 to 15 minutes. A version of the distributed questionnaire is provided in the Appendix.. 

Data analysis

Data analysis was conducted using IBM SPSS Statistics for Windows Version 28.0 (IBM Corp., Armonk, NY). Descriptive statistics were presented as means, frequencies, and percentages to summarize demographic variables and psychological measures. Bivariate analyses were performed using Pearson’s Chi-square and Fisher's exact tests to examine associations between social anxiety levels and various factors. A significance level of *P *< 0.05 was applied to all statistical tests to determine statistical significance.

Ethical consideration

Ethical approval was obtained from the Ethics and Research Committee of the University of Sharjah before data collection. Participation in the study was voluntary, and all participants provided informed consent before completing the questionnaire. To ensure confidentiality and protect participants’ privacy, all questionnaires were distributed online and completed anonymously, with no identifiable personal information collected. Additionally, the survey was piloted on a small sample to verify clarity, comprehension, and appropriateness of the questions, and minor adjustments were made based on participant feedback to enhance understanding and validity.

## Results

Demographics

The sample included 409 adults: 274 (67.0%) females and 135 (33.0%) males. Of the study participants, 248 (61.1%) were aged 18-35 years, while 161 (38.9%) were aged 36 years and older. The mean age was 35.02 years (standard deviation (SD) = 11.98). Non-local Arabs constituted 285 (69.9%) of the study sample, representing the largest group, followed by locals at 69 (16.9%), and non-Arabs at 54 (13.2%). Unmarried individuals comprised 246 (60.1%) of the sample, while married participants represented 163 (39.9%). Our study showed that students constituted the largest portion of the sample, comprising approximately 210 (51.0%) of the respondents. Nearly a third of participants were employed, comprising 126 (31.0%) of the study sample, while 73 (18.0%) were unemployed.

Of the 800 online questionnaires distributed, 409 (51.1%) were completed and submitted.

Association of demographics with social anxiety levels

Table 1 demonstrates that significant correlations exist between social anxiety levels and several demographic factors. Severe-extremely severe levels of social anxiety were noted in 22 (16.3%) of males and 81 (29.6%) of females, indicating a higher prevalence of severe anxiety among the female population (χ²(2, N = 409) = 8.86, *P* = 0.012).

**Table 1:** Association between demographic factors and social anxiety levels. ^*^A *P*-value less than 0.05 indicates a statistically significant association.

Demographic factor	Normal (0-7), *N* (%)	Mild-moderate (8-14), *N* (%)	Severe-extremely severe (15+), *N* (%)	χ² (df)	*P*-value
Gender					
Male	76 (56.3%)	37 (27.4%)	22 (16.3%)	χ²(2) = 8.86	0.012^*^
Female	123 (44.9%)	70 (25.5%)	81 (29.6%)		
Age (years)					
18-35	94 (37.9%)	72 (29.0%)	82 (33.1%)	χ²(2) = 32.49	<0.001^*^
36+	105 (65.2%)	35 (21.7%)	21 (13.0%)		
Ethnicity					
Local Arabs	23 (33.3%)	18 (26.1%)	28 (40.6%)	χ²(4) = 13.41	0.009^*^
Arab (non-local)	144 (50.3%)	76 (26.6%)	66 (23.1%)		
Non-Arabs	32 (59.3%)	13 (24.1%)	9 (16.7%)		
Employment					
Employed	72 (57.1%)	34 (27.0%)	20 (15.9%)	χ²(4) = 39.61	<0.001^*^
Non-employed	51 (69.9%)	16 (21.9%)	6 (8.2%)		
Student	76 (36.2%)	57 (27.1%)	77 (36.7%)		
Marital Status					
Married	105 (64.4%)	38 (23.3%)	20 (12.3%)	χ²(2) = 32.62	<0.001^*^
Not married	94 (38.2%)	69 (28.0%)	83 (33.7%)		
Occupation					
Frontline worker	22 (41.5%)	18 (34.0%)	13 (24.5%)	χ²(2) = 1.25	0.535
Other	177 (49.7%)	99 (25.0%)	90 (25.3%)		

Age also played a significant role (χ²(2, *N* = 409) = 32.49, *P* < 0.001), as 82 (33.1%) of individuals aged 18-35 years exhibited higher social anxiety, whereas 105 (65.2%) of participants aged ≥36 years reported normal anxiety levels.

Ethnicity was significantly associated with social anxiety levels (χ²(4, *N* = 409) = 13.41, *P* = 0.009), with severe anxiety being least prevalent among non-Arabs, reported in 9 (16.7%) of participants. In contrast, Arab locals exhibited the highest tendency to develop severe anxiety, where it affected 28 (40.6%) of individuals, while 66 (23.1%) of Arab non-locals reported severe anxiety. 

It was additionally found that marital status was significantly related to anxiety levels (χ²(2, *N* = 409) = 32.62, *P* < 0.001), as only 20 (12.3%) of married individuals displayed severe social anxiety compared to 83 (33.7%) of unmarried individuals.

Employment status showed a strong association with anxiety severity (χ²(4, *N* = 409) = 39.61, *P* < 0.001), with severe anxiety reported in 77 (36.7%) of students, 20 (15.9%) of employed individuals, and 6 (8.2%) of unemployed participants. 

The analysis suggests that occupation did not influence social anxiety levels in the study population, as the p-value was not statistically significant (*P* = 0.355).

Association between COVID-19-related variables and social anxiety levels

The data revealed interesting correlations between social anxiety levels and several variables related to COVID-19, including infection status, concern about catching the virus, and pre-existing social anxiety diagnoses (Table 2). A history of COVID-19 infection was associated with higher scores on social anxiety-related questions (χ²(2, *N* = 409) = 9.91, *P* = 0.007), with severe to extremely severe levels reported in 60 (31.9%) of infected participants, compared to 43 (19.5%) of uninfected individuals.

**Table 2:** Association between COVID-19-related factors and social anxiety (SA) levels. ^*^A *P*-value less than 0.05 indicates a statistically significant association.

COVID-19-related factor	Normal (0-7), *N* (%)	Mild-moderate (8-14), *N* (%)	Severe-extremely severe (15+), *N* (%)	χ² (df)	*P*-value
Infection with COVID-19					
Yes	78 (41.5%)	50 (26.6%)	60 (31.9%)	χ²(2) = 9.91	0.007^*^
No	121 (54.8%)	57 (25.8%)	43 (19.5%)		
Concern about COVID-19					
Agree	78 (41.7%)	55 (29.4%)	54 (28.9%)	χ²(4) = 11.06	0.026^*^
Neutral	60 (48.4%)	32 (25.8%)	32 (25.8%)		
Disagree	61 (62.2%)	20 (20.4%)	17 (17.3%)		
Pre-COVID-19 SA diagnosis					
Yes	7 (30.4%)	4 (17.4%)	12 (52.2%)	χ²(2) = 9.64	0.008^*^
No	192 (49.7%)	103 (26.7%)	91 (23.6%)		
Lockdown experience					
Yes	180 (47.7%)	100 (26.5%)	97 (25.7%)	χ²(2) = 1.49	0.476
No	19 (59.4%)	7 (21.9%)	6 (18.8%)		

In evaluating concerns regarding COVID-19 infection, individuals who expressed concern exhibited elevated anxiety levels (χ²(4, *N* = 409) = 11.06, *P* = 0.026), with 55 (29.4%) experiencing mild to moderate anxiety and 54 (28.9%) reporting severe to extremely severe anxiety. Notably, participants who were neutral or disagreed with concerns about contracting COVID-19 exhibited lower rates of severe to extremely severe anxiety, with prevalence of 32 (25.8%) and 17 (17.3%), respectively.

Participants with a pre-COVID-19 diagnosis of social anxiety robustly exhibited severe-extremely severe anxiety during the pandemic (χ²(2, *N* = 409) = 9.64, *P* = 0.008), with 12 (52.2%) in this category. Conversely, those without a prior diagnosis displayed a more even distribution across anxiety levels, with 91 (23.6%) experiencing severe anxiety.

Finally, the experience of being in lockdown during the pandemic had no statistically significant impact on social anxiety levels (χ²(2, *N* = 409) = 1.49, *P* = 0.476).

Overall, the data indicated that certain factors related to COVID-19, such as infection status, concern about the virus, and pre-existing social anxiety, all significantly correlated with social anxiety levels, influencing the severity and prevalence of anxiety. Chi-square testing was used to conduct the bivariate analysis of COVID-19-related factors and social anxiety levels.

Association between personality traits and social anxiety levels

The data illustrated significant correlations between social anxiety levels and multiple personality traits, including agreeableness, conscientiousness, emotional stability, and openness to experience (Table 3). Agreeableness presented a significant relationship with social anxiety levels (χ²(4, *N* = 409) = 17.76, *P* = 0.001), where 21 (41.2%) of highly agreeable participants displayed severe social anxiety. In contrast, 8 (17.0%) of those who scored low on agreeableness exhibited severe levels of social anxiety.

**Table 3:** Association between the five personality traits and levels of social anxiety. ^*^A *P*-value less than 0.05 indicates a statistically significant association.

Personality trait	Social anxiety level	Normal (0-7), *N* (%)	Mild-moderate (8-14), *N* (%)	Severe-extremely severe (15+), *N* (%)	*P*-value	χ² (df)
Extraversion	Low	22 (57.9%)	10 (26.3%)	6 (15.8%)	0.366	
	Normal	171 (48.4%)	92 (26.1%)	90 (25.5%)		
	High	6 (33.3%)	5 (27.8%)	7 (38.9%)		
Agreeableness	Low	32 (68.1%)	7 (14.9%)	8 (17.0%)	0.001^*^	17.76
	Normal	144 (46.3%)	93 (29.9%)	74 (23.8%)		
	High	23 (45.1%)	7 (13.7%)	21 (41.2%)		
Conscientiousness	Low	29 (76.3%)	5 (13.2%)	4 (10.5%)	<0.001^*^	26.75
	Normal	142 (50.9%)	74 (26.5%)	63 (22.6%)		
	High	28 (30.4%)	28 (30.4%)	36 (39.1%)		
Emotional Stability	Low	34 (69.4%)	9 (18.4%)	6 (12.2%)	<0.001^*^	19.63
	Normal	148 (49.0%)	81 (26.8%)	73 (24.2%)		
	High	17 (29.3%)	17 (29.3%)	24 (41.4%)		
Openness to experience	Low	39 (57.4%)	16 (23.5%)	13 (19.1%)	0.013^*^	12.60
	Normal	149 (50.0%)	77 (25.8%)	72 (24.2%)		
	High	11 (25.6%)	14 (32.6%)	18 (41.9%)		

As shown in Table 3, individuals with high conscientiousness were more likely to report severe social anxiety, with 36 (39.1%) affected, compared to those with low conscientiousness, of whom 4 (10.5%) experienced severe anxiety. This association was statistically significant (χ²(4, *N* = 409) = 26.75, *P* < 0.001).

Emotional stability was also closely related to social anxiety levels (χ²(4, *N* = 409) = 19.63, *P* < 0.001). Among participants with high emotional stability, 24 (41.4%) experienced severe social anxiety, whereas 34 (69.4%) of those with low emotional stability exhibited normal levels of social anxiety.

Openness to experience showed a further significant correlation with social anxiety levels among participants (χ²(4, *N* = 409) = 12.60, *P* = 0.013). Among those with high openness, 18 (41.9%) experienced severe social anxiety, whereas only 13 (19.1%) of participants with low openness were affected.

With a *P*-value of 0.366, there was no significant association between social anxiety levels and the extraversion personality trait, and therefore, the null hypothesis could not be rejected.

**Figure 1:**
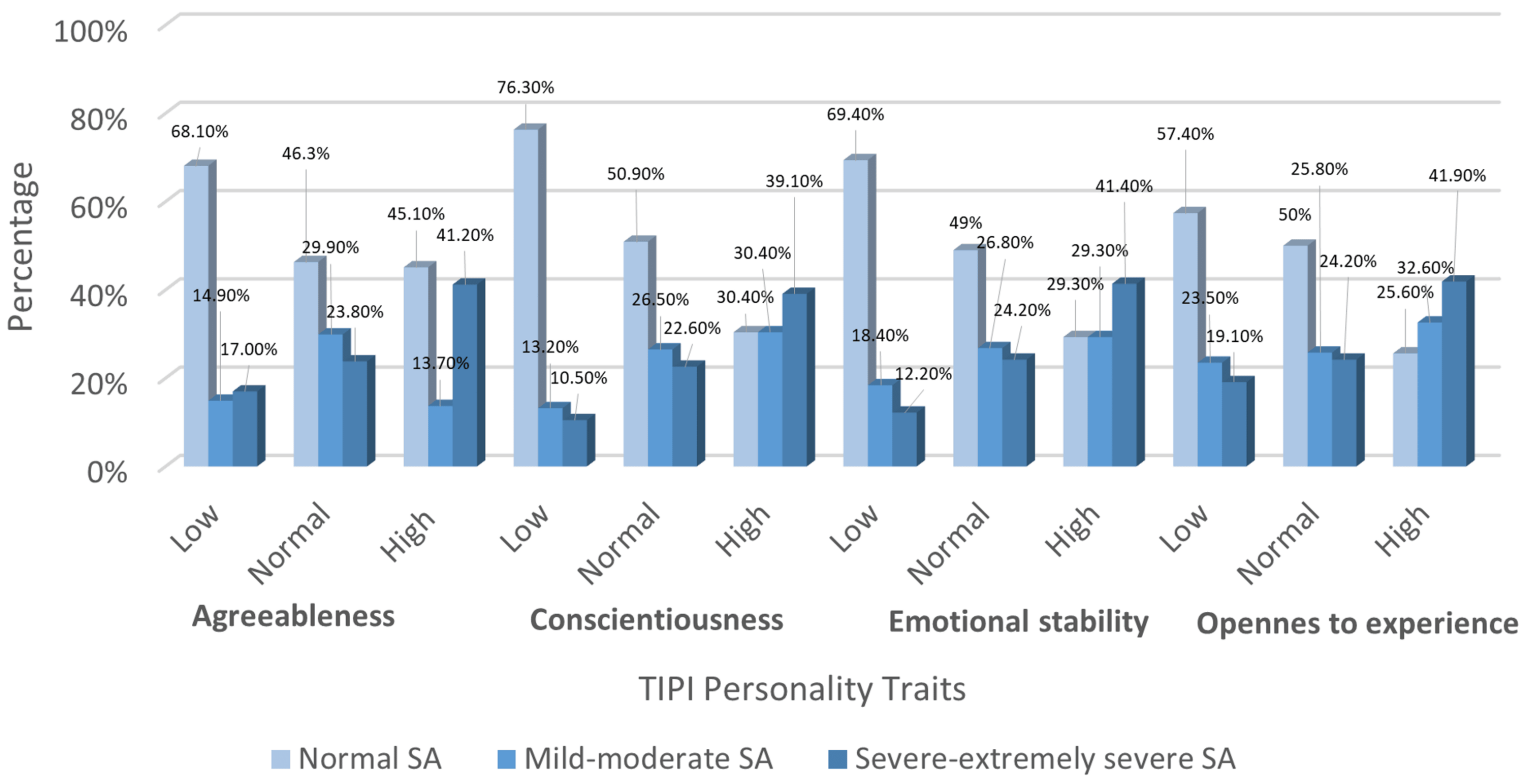
Association between social anxiety levels and agreeableness, conscientiousness, emotional stability, and openness to experience. TIPI, Ten-Item Personality Inventory

Overall, the data suggest that several personality traits significantly influence social anxiety levels. This association is illustrated in Figure 1. Chi-square testing was used to conduct the bivariate analysis of all personality traits with social anxiety, except extraversion, where Fisher’s exact test was applied due to small expected cell counts in the *High Extraversion* group.

## Discussion

This study aimed to assess the prevalence of social anxiety among adults in the UAE following the COVID-19 lockdown and to identify key demographic, personality, and pandemic-related risk factors. The findings reveal significant associations between social anxiety severity and multiple factors, highlighting the complexity of post-pandemic mental health challenges.

A substantial proportion of the study population exhibited severe social anxiety, with notable differences based on demographic characteristics. Female participants demonstrated higher levels of severe social anxiety compared to males, a trend consistent with existing literature suggesting that women are more susceptible to anxiety-related disorders due to hormonal, psychological, and sociocultural factors [13]. Additionally, younger adults (18-35 years) displayed significantly higher social anxiety levels than older participants. This observation aligns with a previous study by Singh et al., indicating that young adults may be more affected by the abrupt social disruptions caused by lockdowns and the increased reliance on digital communication during the pandemic, which could exacerbate social avoidance behaviors [14].

Marital status and employment were also influential factors, with unmarried individuals and students reporting higher social anxiety levels. The protective role of social relationships is well-documented in anxiety research, and the lack of a stable social support system in unmarried individuals may contribute to heightened anxiety levels [15]. Similarly, students, who constituted a significant proportion of the sample, may have experienced increased social stress due to disrupted academic environments, uncertainty regarding future career prospects, and limited in-person interactions post-lockdown [16].

The analysis found no significant association between occupation and social anxiety levels; both front-line and non-front-line workers exhibited similar distributions of mild, moderate, and severe anxiety. This finding contrasts with previous research indicating that front-line workers are more susceptible to anxiety and depressive disorders due to increased exposure to stressors during the COVID-19 pandemic. For instance, a meta-analysis by Saragih et al. found that front-line healthcare workers had a higher prevalence of anxiety symptoms compared to non-front-line staff [17]. The discrepancy between these findings and existing literature may be attributed to multiple factors. One possible explanation is that, in the UAE, front-line workers may have received substantial institutional support, including mental health resources, financial stability, and structured workplace interventions that mitigated anxiety levels [18]. Additionally, cultural and societal factors might play a role, as the region's strong familial and community support systems could have buffered the psychological burden on front-line workers [19]. Another consideration is that the general public, particularly those in non-frontline jobs, may have faced unique stressors, such as job insecurity, social isolation, and financial concerns, which could have contributed to comparable levels of social anxiety [20]. Finally, differences in sample characteristics and the self-reported nature of anxiety assessments may have influenced the results, leading to variations from global findings.

The impact of COVID-19-related variables on social anxiety was also evident. Participants who had been infected with COVID-19 or expressed concerns about contracting the virus exhibited higher levels of social anxiety. This supports previous findings that link health-related anxiety with social withdrawal and increased fear of judgment in social settings [21]. Additionally, individuals with a prior SAD diagnosis demonstrated markedly higher social anxiety levels post-lockdown, suggesting that pandemic-induced stressors may have exacerbated preexisting anxiety disorders. Despite the presented data showing no statistically significant association between experiencing lockdown during the COVID-19 pandemic and social anxiety levels, studies have shown increased SAD levels among individuals who have been in lockdown, underscoring the psychological impact of prolonged social isolation [22].

Personality traits played a significant role in determining social anxiety severity. High levels of conscientiousness, emotional stability, agreeableness, and openness to experience were significantly correlated with severe social anxiety. These findings suggest that individuals with heightened self-awareness and sensitivity to social interactions may be more prone to experiencing distress in post-pandemic social settings [23].

A notable finding of this study is the positive relationship between emotional stability and social anxiety. While research on this association is limited, a prior study among medical and humanities students reported the opposite pattern, with higher emotional stability linked to lower social anxiety. This discrepancy suggests that the relationship may be influenced by cultural or contextual factors. Although emotionally stable individuals are generally calm and less reactive to stress, in contexts where social image and behavior are highly salient, such as in the UAE, they may engage in heightened self-monitoring to maintain control in social interactions. In collectivist cultures that emphasize social harmony and reputation, this increased self-awareness may paradoxically elevate social anxiety. These findings indicate that the protective effects of emotional stability on anxiety may vary depending on cultural and social context [24].

Interestingly, extraversion did not show a significant association with social anxiety levels, which contrasts with some prior studies suggesting that extraverts typically report lower anxiety in social environments. For instance, research has indicated that individuals with low levels of extraversion may feel more anxious in social situations [25]. Additionally, studies have found that many disorders, including SAD, are associated with low levels of extraversion [26]. This discrepancy may be influenced by cultural factors unique to the UAE, where social interactions often carry high societal expectations and norms. 

Given the cross-sectional research design employed in this study, establishing causal relationships between the examined variables remains a limitation. Future longitudinal studies incorporating subject follow-ups are recommended to investigate the associations between COVID-19 experiences, personality traits, and social anxiety. Additionally, incorporating participants from diverse socioeconomic backgrounds may enhance the generalizability and validity of the findings. Moreover, the study was limited to individuals aged 18 and above, preventing an assessment of social anxiety among younger adolescents who the pandemic may have significantly impacted. Furthermore, only bivariate analysis (chi-square tests) was performed, without applying multivariate regression to adjust for potential confounders, such as age, gender, and prior mental health conditions. Another limitation is the use of snowball sampling, which, while beneficial in reaching participants more inclined to discuss mental health issues, may have introduced selection bias, as the sample might not be fully representative of the broader UAE population.

The study underscores the necessity for targeted mental health interventions to address post-pandemic social anxiety in the UAE, highlighting the complex interplay between demographic, psychological, and pandemic-related factors in shaping social anxiety among adults. Given the identified risk factors, interventions should focus on providing tailored support to high-risk groups, such as young adults, women, students, and individuals with preexisting anxiety disorders. Awareness programs emphasizing coping strategies, social reintegration, and psychological resilience can be instrumental in mitigating the long-term effects of social anxiety. Additionally, integrating mental health support into educational institutions and workplaces may facilitate early detection and intervention, ultimately improving the well-being of affected individuals. These findings offer valuable insights into the post-pandemic mental health landscape, providing a foundation for developing evidence-based interventions to support individuals struggling with social anxiety.

## Conclusions

This study underscores the urgent need to address social anxiety in the UAE, particularly among high-risk groups such as young adults, women, students, and individuals with preexisting anxiety. Findings support the need for integration of screening programs into primary care and university settings to detect and manage social anxiety early. Policy and practice efforts should prioritize accessible mental health services and culturally tailored interventions to promote social reintegration and overall mental well-being. Future research should explore longitudinal trends and effective public health strategies to mitigate the long-term effects of social anxiety in the UAE.

## Appendices

Appendix 

**Table 4:** English version of the social anxiety questionnaire DASS-21: Depression, Anxiety, and Stress Scale - 21 Items TIPI: Ten-Item Personality Inventory

Section Name	Item Number	Questions / Instructions	Choices
Demographics	D1	Are you living/resident in the UAE?	Yes
			No (If No → Survey ends)
	D2	Sex	Male
			Female
	D3	Age Group	18–35
			36–50
			51–64
			65+
	D4	Nationality	Local
			Other (please specify)
	D5	Marital Status	Single, never married
			Married
			Widowed
			Divorced
	D6	Diagnosed with social anxiety disorder before COVID-19 pandemic?	Yes
			No
	D7	Employment Status	Employed
			Unemployed
			Retired
			Student
	D8	Occupation	Frontline worker
			Other (please specify)
COVID-19	C1	Have you been infected with COVID-19?	Yes
			No
	C2	Do you know someone close who got infected?	Yes
			No
	C3	Were you in lockdown during COVID-19?	Yes
			No
	C4	When did you return to work/duties?	During lockdown
			After lockdown
			Still working online
			Unemployed/Retired
	C5	Worried about catching COVID-19 on commute?	Strongly Agree
			Agree
			Neutral
			Disagree
			Strongly Disagree
	C6	Worried about COVID-19 infecting your family?	Strongly Agree
			Agree
			Neutral
			Disagree
			Strongly Disagree
DASS-21	A1	Dryness of mouth	Never
			Sometimes
			Often
			Almost always
	A2	Breathing difficulty (e.g., rapid, no exertion)	Never
			Sometimes
			Often
			Almost always
	A3	Shakiness (e.g., legs giving way)	Never
			Sometimes
			Often
			Almost always
	A4	Relief when anxious situations ended	Never
			Sometimes
			Often
			Almost always
	A5	Faintness	Never
			Sometimes
			Often
			Almost always
	A6	Noticeable sweating without heat/exertion	Never
			Sometimes
			Often
			Almost always
	A7	Felt scared for no good reason	Never
			Sometimes
			Often
			Almost always
TIPI	P1	Social, lively	Strongly Agree
			Agree
			Neutral
			Disagree
			Strongly Disagree
	P2	Critical, argumentative	Strongly Agree
			Agree
			Neutral
			Disagree
			Strongly Disagree
	P3	Reliable, self-controlled	Strongly Agree
			Agree
			Neutral
			Disagree
			Strongly Disagree
	P4	Distressed, easily upset	Strongly Agree
			Agree
			Neutral
			Disagree
			Strongly Disagree
	P5	Approachable	Strongly Agree
			Agree
			Neutral
			Disagree
			Strongly Disagree
	P6	Uncommunicative, quiet	Strongly Agree
			Agree
			Neutral
			Disagree
			Strongly Disagree
	P7	Comforting, warm	Strongly Agree
			Agree
			Neutral
			Disagree
			Strongly Disagree
	P8	Disorganized, careless	Strongly Agree
			Agree
			Neutral
			Disagree
			Strongly Disagree
	P9	Calm, emotionally stable	Strongly Agree
			Agree
			Neutral
			Disagree
			Strongly Disagree
	P10	Ordinary, uncreative	Strongly Agree
			Agree
			Neutral
			Disagree
			Strongly Disagree
